# Current Cigarette Smoking and Its Predictors among School-Going Adolescents in East Africa: A Systematic Review and Meta-Analysis

**DOI:** 10.1155/2019/4769820

**Published:** 2019-05-08

**Authors:** Nega Tezera, Aklilu Endalamaw

**Affiliations:** Department of Pediatrics and Child Health Nursing, School of Nursing, College of Medicine and Health Sciences, University of Gondar, Gondar, Ethiopia

## Abstract

**Background:**

In developing countries, tobacco smoking has its own contribution to the burden of noncommunicable causes of morbidity and mortality. Studies estimated the burden of cigarette smoking among school-going adolescents in different geographical areas of East Africa. However, due to discrepancies found among those different findings, there is no representative data about the burden of smoking in the continent.

**Objectives:**

This systematic review and meta-analysis aimed to assess the pooled prevalence of current cigarette smoking and its associated factors among school-going adolescents in East Africa.

**Methods:**

PubMed, Google Scholar, and the Web of Science Library were searched to access included articles. A weighted inverse-variance random-effects model was used to estimate the prevalence of current cigarette smoking. Variations in the pooled estimates of the prevalence were adjusted through subgroup analysis according to the specific country, where the study was conducted. Funnel plot and Egger's regression test were used to check publication bias. STATA version 14 statistical software was used for meta-analysis.

**Results:**

A total of 26,875 school-going adolescents were included. The pooled prevalence of current cigarette smoking among school-going adolescents in East Africa was found to be 9.02% (95%CI: 6.34-11.70). Based on the subgroup analysis, current cigarette smoking among school-going adolescents was estimated at 9.8% in Kenya, 7.72% in Ethiopia, 10.83% in Uganda, 13.6% in Sudan, and 4% in Tanzania.

**Conclusions:**

This meta-analysis revealed that the prevalence of current cigarette smoking is increasing among school-going adolescents in East Africa. Therefore, countries have to realize sale prevention policies, establishing and/or strengthening antismoking campaigners designed for school-going adolescents, and providing training for teachers to be antismoking campaigners.

## 1. Background

Tobacco smoking is one of the notable public health threats killing more people than HIV/AIDS, tuberculosis, and malaria in the world. It is projected that by the end of 2030, the number of deaths would increase by 8 million per year and could account for 10% of the total global mortality by 2020, killing nearly one billion people [1, 2].

Moreover, in developing countries, tobacco smoking has been responsible for an increasing burden of noncommunicable causes of deaths and diseases [3, 4]. Evidence shows that sustained tobacco use leads to different health consequences due to exposure to nicotine [5, 6]. For instance, major depression, anxiety, periodontal, peripheral, vascular, and heart disease, stroke, chronic obstructive pulmonary disease, pneumonia, and lung cancers have been recorded among cigarette smokers [7–10]. Moreover, smoking also exposed smokers to many risky behaviors, like fighting and unprotected sex [11].

In the world, many adults mostly begin to smoke at the age of adolescence, the period at which young people are usually exposed to tobacco experimentation and/or smoking their first cigarettes [5, 6]. Today, more than 600,000 and 3 million middle- and high-school students have experienced smoking cigarettes, respectively [12, 13]. Worldwide, sustainable development goals have been planned to reduce cigarette smoking by implementing different strategies like advertising restrictions, changing policies, and implementing school health education and community advocacy programs. For example, a school-based program can reduce short-term smoking prevalence in the range of 30%-70%. This program can save approximately between 2,000 and 20,000 US$ per QALY in contrast with the total cost needed for other interventions, which ranges from 16,400 to 580,000 US$ [14]. Despite the implementation of adolescent-focused strategies, cigarette smoking was reported as 51% in southern Brazil [15], 10.7% to 14.7% in Taiwan [16], and 15.5% in Africa [17].

The easy accessibility of tobacco, traditions and moderate pricing, peer and family influence, and tobacco company advertisements and promotional activities [18] are some of the predisposing factors to cigarette smoking among adolescents. Besides, male sex, age, type of school, alcohol use, no exposure to antismoking media messages, lack of attention at schools, exposure to the movies with smoker actors, and smoker teachers [19–29] are identified associated factors from previous studies.

Investing on the prevention of young people from smoking is the sole strategy to mitigate the health consequences [30]. According to research, East Africa falls in the range of 4% to 32% by the magnitude of school-going adolescent smokers although there have been no continent-based representative data. Therefore, this study aimed to estimate the overall prevalence of current cigarette smoking and its associated factors among school-going adolescents in East Africa in order to contribute to a continent-based policy formulation.

## 2. Method and Materials

### 2.1. Reporting

The result of this review was reported in accordance with the Preferred Reporting Items for Systematic Reviews and Meta-Analyses guideline (PRISMA) [31] (PRISMA checklist), and it is registered in the Prospero database (PROSPERO 2018 [CRD42018096283]) accessed at https://www.crd.york.ac.uk/prospero/#searchadvanced.

### 2.2. Information Source and Search Strategy

We searched all published studies reporting with the prevalence and/or associated factors of cigarette smoking among school-going adolescents in East African countries.

PubMed, Google Scholar, and Web of Science library were searched. Search strategies were established using “AND” and/or “OR” Boolean operators. The search strategy for PubMed was Adolescent [MeSH Terms] OR youth OR children OR young OR AND predictors [MeSH Terms] OR risk factors OR enablers OR determinants OR covariates OR associated factors OR predisposing factors OR factors AND prevalence [MeSH Terms] OR burden OR incidence OR magnitude OR epidemiology AND tobacco smoking [MeSH Terms] OR cigarette smoking OR smoking OR cigar OR substance use OR substance abuse AND East Africa.

### 2.3. Study Inclusion Criteria

Studies were included if and only if they fulfilled the following criteria.

Study design: the search was designed to access cross-sectional, cohort, and case-control studies. Participants: this systematic review considered school-going adolescents or young students.

Studies which reported the prevalence and/or at least one associated factor were included.

Language: studies published in English were included.

### 2.4. Exclusion Criteria

Studies conducted other than adolescent students, editorial comments, conference proceedings, and qualitatively described works were excluded.

### 2.5. Quality Assessment Tool

Two critical appraisers (NT, AE) autonomously assessed the quality of the study using the Joanna Briggs Institute Meta-Analysis of Statistics Assessment and Review Instrument (JBI-MAStARI) [32]. The JBI critical appraisal checklist tool for cross-sectional studies which consists of eight criteria was used. These are (1) defined criteria for inclusion, (2) described study subject and the setting, (3) exposure measured in a valid and reliable method, (4) stated objective and standard criteria for measurement, (5) identified confounding factors, (6) stated strategies for confounding factors, (7) outcome measurement, and (8) appropriate statistical analysis used. Studies which scored 50% and above of the quality assessment criteria were considered low-risk bias.

### 2.6. Data Abstraction Tool and Process

Two authors (N.T & A.E) independently reviewed abstracts and full-text articles, using a preestablished data abstraction tool. Data were extracted by Microsoft Office Excel 2007. The name of the first author, the country where research was conducted, study design, study setting, the age of study participants, the year of study, and sample size were extracted. Whenever disagreement happened, it was resolved by discussion and/or repeating the procedure.

### 2.7. Measurement of Outcome

Current cigarette smoking was defined as “having smoked at least once in the past 30 days.”

The prevalence of cigarette smoking was determined by dividing who were smoking at the moment by the overall participants of the study.

### 2.8. Statistical Analysis

A weighted inverse-variance random-effects model [33] was used to estimate the prevalence of smoking. The variation in the pooled estimates of the prevalence was adjusted through subgroup analysis according to the country, where the included studies were conducted. Heterogeneity across studies was considered low, moderate, and high when an I^2^ statistic value denoted 25%, 50%, and 75%, respectively [34]. Funnel plot and Egger's regression test were used to check publication bias [35]. STATA version 14 statistical software was used for meta-analysis. The sensitivity analysis was also applied to see the impact of every single study on the overall estimate. Factors significantly associated with the reported adjusted odds ratio were described qualitatively.

## 3. Results

### 3.1. Literature Search and Selection of Eligible Material

**Figure 1:**
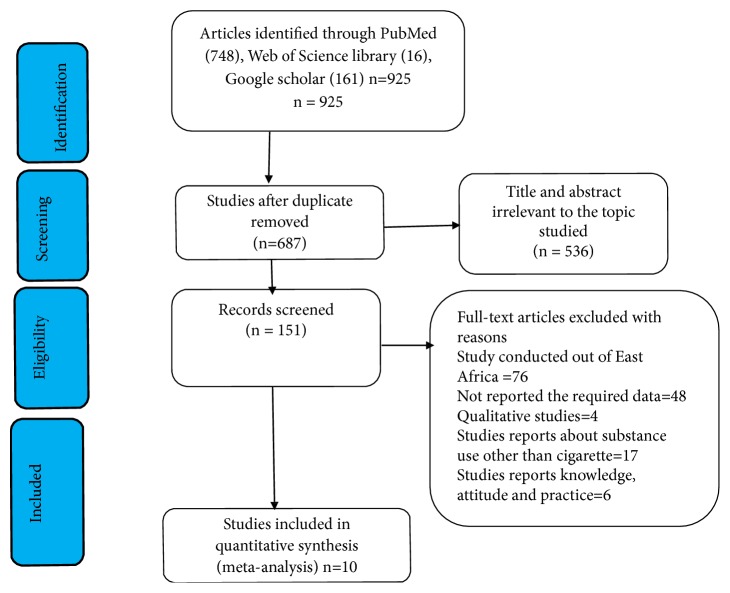
PRISMA flow diagram of identification and selection of studies for this systematic review and meta-analysis.

A total of 925 original articles were identified from PubMed, Google Scholar, and the Web of Science Library. Out of all identified studies, 238 were found duplicated. Out of 687 studies, 536 were excluded based on the screening of titles and abstracts. Again, 76 studies were excluded owing to being studies conducted outside East African countries. Finally, ten articles were used for meta-analysis (Figure 1).

### 3.2. Characteristics of Included Studies

Ten studies were conducted in East African countries between 2003 and 2014. Of these, four were in Ethiopia [21–23, 36], three in Uganda [19, 28, 29], and one each in Kenya [25], Sudan [24], and Tanzania [26]. All of the studies accessed through the search were cross-sectional. Sample sizes ranged from 651 [21] to 12,378 [25] (Table 1).

### 3.3. Quality of Included Studies

Based on the JBI critical appraisal checklist for analytical cross-sectional studies, all of the papers had no methodological defect and significant risk of bias. Hence, no article was excluded from the meta-analysis.

### 3.4. Meta-Analysis

#### 3.4.1. Prevalence of Current Cigarette Smoking

**Figure 2:**
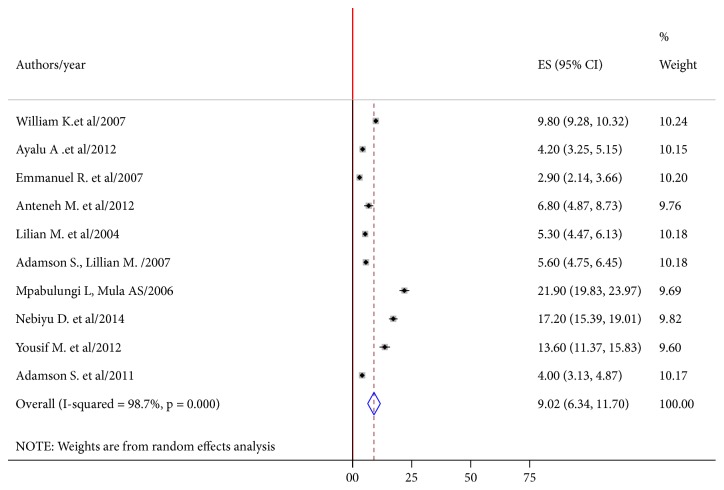
Forest plot of the pooled estimates (ES) cigarette smoking among school-going adolescents. The midpoint and the length of each segment indicated prevalence and a 95% CI, whereas the diamond shape showed the combined prevalence of all studies.

The combined prevalence of current cigarette smoking was found to be 9.02% (95% CI: 6.34-11.70; I2 =98.7%,* P* value<0.001) among school adolescents in East Africa (Figure 2).

#### 3.4.2. Subgroup Analysis

**Figure 3:**
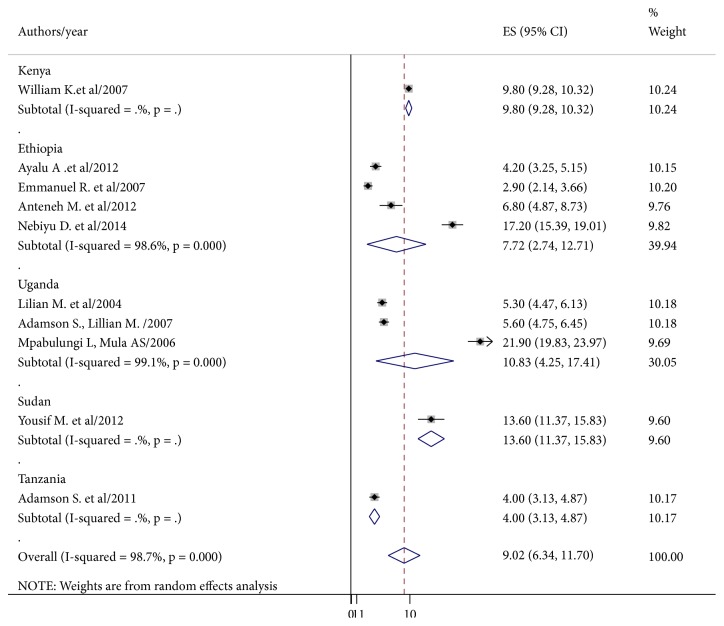
The forest plot showed the subgroup analysis of the prevalence (ES) of cigarette smoking among school-going adolescents. The midpoint and the length of each segment indicated prevalence and a 95% CI, whereas the diamond shape showed the combined prevalence of all studies.

Subgroup analysis based on the country, where the studies were conducted, was done. The result revealed that the prevalence of current cigarette smoking among school-going adolescents was 9.8% in Kenya, 7.72% in Ethiopia, 10.83% in Uganda, 13.6% in Sudan, and 4% in Tanzania (Figure 3).

#### 3.4.3. Sensitivity Analysis

The sensitivity analysis showed that there was little change that could not affect the overall outcome estimate too much (Table 2).

#### 3.4.4. Publication Bias

**Figure 4:**
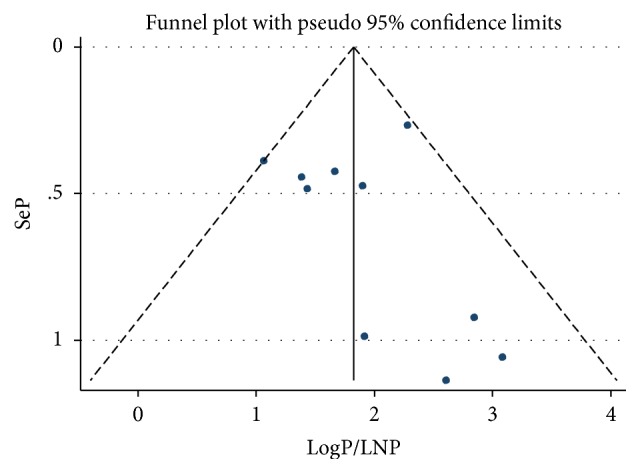
Funnel plot vertical lines estimate the effect size, whereas a diagonal line measures the precision of individual studies with a 95 % confidence limit.

A funnel plot was employed to see the symmetry of publications (Figure 4), and Egger's test showed no publication bias (*P* value=0.604).

#### 3.4.5. Predisposing Factors to Current Cigarette Smoking

According to the review, ten predictors were found to be significantly associated with cigarette smoking among school-going adolescents (Table 3).

## 4. Discussion

Adolescence is a critical period of a developmental milestone, in which a significant number of smokers are initiated to smoke [5] and the gateway to other risky behaviors, like drug use. This systemic review and meta-analysis was conducted to estimate the pooled prevalence and associated factors of current cigarette smoking among school-going adolescents in East Africa. The pooled prevalence of the practice among the adolescents in East Africa was 9.02% (95%CI: 6.34-11.70). This finding is congruent to those of studies done in Botswana (10%) [37] and Brazil (10.7%) [38]. In this setting, many people were living in the category of low socioeconomic status. Smoking prevalence was found to be higher among low socioeconomic status groups compared with their counterpart [39]. On the other hand, the current finding is higher than the prevalence reported in Bangladesh (2%) [37], Iran (2.7%) [38], Nigeria (4.2%) [39], and China (7.93%) [40]. This could be due to the fact that there were either ban or only partial ban policy implementations on cigarette sale promotion [41, 42] and owing to, perhaps, more exposure to tobacco advertisements [43, 44] in East African countries. In addition, the absence of school-based antismoking campaigners including trained teachers, lacking tobacco prevention initiatives, like social competence and influence interventions in schools, and the nonexistence of family-based interventions deserve blames. The implementation of these interventions helps adolescents to reduce experimentation with smoking by 16% to 32% [45]. Besides, lack of cigarette access restriction and sale prevention policies and smoking cessation program contributes to the high proportion of smoker adolescents in East African countries [46–48].

The finding of the current study is lower than those of studies done in Brazil 13.0% [49], Pakistan (13.7%) [50], and China 14.12% [51]. This difference might be due to the fact that some indigenous cultures allow adolescents to access cigarette or tobacco as well as differences in religious teachings.

The subgroup analysis showed that the prevalence of the current cigarette smoking in Sudan (13.6%) was similar to that of Uganda (11.21%) and Kenya (9.8 %). A large proportion of the adolescents lived in low-income countries of northeast Africa. As a result, tobacco companies' shifted their production to this region because of the existence of cheap child labor for tobacco cultivation and picking and the availability of large-scale tobacco farms. However, the result was higher than that of Tanzania (4%) and Ethiopia (7.72%). The possible explanation for this variation may be the socioeconomic status of individual, communities differences in the social norms, high production of tobacco at local community level as small-scale farming for income generation, sample size differences, and incoherent tobacco control and prevention policies of the countries.

In East African countries, having peers who smoke tobacco was significantly associated with being smokers. This result was consistent with those of Pakistan, Turkey, and Saudi Arabia which were [50, 52, 53], respectively. This could be because early adolescents psychologically consider themselves as unique and invulnerable, they experience a sense of separation or independence as a developmental task, and they spend plenty of their time with many peers. In due course, this “personal fable” leads to detachment of adolescents from their family ties or parental values.

Male sex [54–56] was a predisposing factor to cigarette smoking. This could be related to the fact that males are not socially sanctioned as females. As well, having multiple partners is a prescribed and socially accepted and expected role of the male in some East African countries.

Having family members who smoked [50, 52, 53, 57] had also predisposed school-going adolescents to the habit. This may be because families are the primary sources of reinforcement, and adolescents whose fathers were in the lowest occupational groups were two times as likely to those whose fathers occupying the highest occupational status.

Exposure to movies with smoking actors, having some pocket money, and not discussing in class about the dangers of smoking were notable predisposing factors [52]. This is because health education and antismoking sessions were not incorporated in the primary and secondary school curricula. Due to this, adolescents could not improve their knowledge, modify their attitude, and decide to quit smoking.

## 5. Limitation of This Study

Though this review is the first systematic review and meta-analysis done in East Africa, it has its own limitation. No studies were found in all East African countries. Besides, we may missed studies conducted other than in English language.

## 6. Conclusions

The finding of this meta-analysis revealed that the prevalence of cigarette smoking was increasing among school-going adolescents in East Africa at the moment, and various risk factors are responsible for the exposure. Therefore, countries have to realize sale prevention policies, implement antismoking campaigners, emphasize health hazard adverts about cigarettes, and integrate health education on cigarettes in youth-friendly services.

## Figures and Tables

**Table 1:** Characteristics of systemic review and meta-analysis articles (n=10).

Author/Year	Study Area	Study design	Sample size	Prevalence (95% CI) of cigarette smoking	Quality
William K.et al/2007[26]	Kenya	Cross-sectional	12378	9.8	Low risk
Ayalu A.et al/2012[21]	Ethiopia	Cross-sectional	1721	4.2	Low risk
Emmanuel R. et al/2007[23]	Ethiopia	Cross-sectional	1868	2.9	Low risk
Anteneh M. et al/2012[22]	Ethiopia	Cross-sectional	651	6.8	Low risk
Lilian M. et al/2004[20]	Uganda	Cross-sectional	2789	5.3	Low risk
Adamson S., Lillian M. /2007[28]	Uganda	Cross-sectional	2789	5.6	Low risk
Mpabulungi L, Mula AS/2006[27]	Uganda	Cross-sectional	1528	21.9	Low risk
Nebiyu D. et al/2014[24]	Ethiopia	Cross-sectional	1673	17.2	Low risk
Yousif M. et al/2012[25]	Sudan	Cross-sectional	910	13.6	Low risk
Adamson S. et al/2011[29]	Tanzania	Cross-sectional	1947	4	Low risk

**Table 2:** Sensitivity analysis of the prevalence of cigarette smoking among school-going adolescents.

Study omitted	Prevalence of current cigarette smoking (95%CI )
William K.et al/2007	8.94 (6.0,11.8)
Ayalu A.et al/2012	9.57(6.6,12.5)
Emmanuel R. et al/2007	9.71(6.8,12.5)
Anteneh M. et al/2012	9.26(6.3,12.1)
Lilian M. et al/2004	9.4(6.4,12.5)
Adamson S, Lillian M. /2007	9.4(6.3,12.4)
Mpabulungi L, Mula AS/2006	7.6(5.2,9.7)
Nebiyu D. et al/2014	8.1(5.5,10.7)
Yousif M. et al/2012	8.1(5.0,11.1)
Adamson S. et al/2011	8.5(5.7,11.3)
Combined	9.6(6.6,12.5)

**Table 3:** Associated factors of current cigarette smoking among school adolescents in East Africa.

Sr. No.	Sociodemographic related factors	Associated factors
1	Sex of students	Male had higher odds of smoking compared to female
2	Age of students	Higher age was found to be a significant predictor of smoking
3	Peer influence	Having peer friends who smoke cigarette was found to be a significant predictor of smoking
4	Perception of risk of smoking	Harmful perception about risk of smoking was negatively associated with smoking cigarette
5	Family's substance use	History of substance use among family was positive associated factor with cigarette smoking of adolescent
6	Sibling's substance use	Sibling's substance use was a significant predictor of substance use
7	Owning an item with a cigarette brand log	Current cigarette smokers were more likely to have an item with a cigarette logo compared to cigarette nonsmokers, OR 2.0 (95% CI: 1.1-3.5).
8	Exposure to movie with actors smoking	Adolescents who were exposed to movies whose actors are smokers were seen 2.8 times more likely to use tobacco (AOR = 2.84, 95% CI 1.703–11.116)
9	Exposure to antismoking media message	Adolescents who were not exposed to antismoking media messages on television, radio, newspaper or magazines were seen more likely to use tobacco than those who were exposed (AOR = 4.43, 95% CI 1.838–7.13)

## Data Availability

All data are available within the paper.

## Abbreviations

JBI: Joanna Briggs Institute
HIV/AIDS: Human immunodeficiency virus/acquired immunodeficiency syndrome
PRISMA-P: Preferred items for systemic reviews and meta-analysis protocol.
